# Investigating the Effect of Repeated High Water Pressure on the Compressive and Bond Strength of Concrete with/without Steel Bar

**DOI:** 10.3390/ma14030527

**Published:** 2021-01-22

**Authors:** Ahmad Aki Muhaimin, Mohamed Adel, Kohei Nagai

**Affiliations:** 1 Department of Civil Engineering, The University of Tokyo, Tokyo 113-8654, Japan; ahmadaki@iis.u-tokyo.ac.jp; 2 Institute of Industrial Science, The University of Tokyo, Tokyo 113-8654, Japan; nagai325@iis.u-tokyo.ac.jp; 3 Department of Structural Engineering, Faculty of Engineering, Cairo University, Cairo 12613, Egypt

**Keywords:** deep-sea structures, high water pressure, concrete cylinder, compressive strength, bond strength, failure damage

## Abstract

The application of reinforced concrete for permanent and temporary deep ocean structures has recently become more prevalent; however, the static and dynamic effects of high water pressure on concrete remain unexplored. This paper investigates the influence of high water pressure (60 MPa) on four series of concrete cylinders with and without an embedded steel bar under sustained and cyclic loading conditions. The residual compressive strength, bond strength, and associated evolution of surface and internal damage are evaluated after exposing concrete cylinders to a water pressure of 60 MPa. The first series is exposed to sustained water pressure for 7 and 60 days, while the other series is tested under repeated water pressure for 10, 20, 30, 60, and 150 cycles. The results reveal that residual compressive strength falls immediately by 16% within 7 days of sustained high water pressure, but the strength then remains stable up to 60 days. Under repeated high water pressure, residual compressive strength gradually reduces by up to 40% until 60 cycles, after which it remains reasonably stable until 150 cycles as crack propagation is arrested at a certain depth within the concrete cylinders. The bond strength between the steel bar and matrix is observed to decrease considerably under repeated cycles of 60 MPa water pressure up to 26%. The damage gradually propagates at the matrix/steel bar interface under the repeated water pressure, resulting in a reduction in residual pullout capacity.

## 1. Introduction

There has recently been growing interest in the utilization of concrete structures at great depths for the purpose of deep ocean exploration [1,2,3,4,5,6,7,8]. Concrete is the most widely used building material for structures in shallow waters up to 1000 m deep, including dams, bridge piers, offshore structures, and marine petroleum platforms, where the hydraulic pressure is in the range of 0–10 MPa [9,10,11,12,13]. Such structures are under static and hydrodynamic loading as a result of the water pressure, resulting in complicated stress states [14,15]. Further, water readily penetrates the concrete under higher pressure, leading to degradation of its internal mechanical properties. Although the static and fatigue behavior of concrete are well understood in shallow seas [16,17,18,19,20,21,22,23], the effect of the high water pressures experienced in deep seas has still not yet been considered in practice [24,25,26]. Thus, the response of concrete to sustained and repeated high water pressure, including its effect on uniaxial compression strength and the bond strength with steel bars, needs to be investigated together with its damage evolution.

The focus of deep ocean exploration is currently the discovery of animal and/or plant life (ecosystems), plate tectonics, and the investigation of minerals and soil structures on the seabed at depths greater than 1000 m. At such depths, many physical, biological, geological, chemical, and archaeological aspects of the ocean remain a mystery and are a challenge for scientists [1,2,3,4,5,6,7,8]. There is a need for permanent and/or temporary structures in the deep ocean from which to undertake the mining of rare-earth elements, monitoring of ecosystems, and exploring of the seabed. Concerning that, there will be a high demand for the construction of deep-sea stations for deep ocean exploration in the near future. Concrete is considered the most suitable construction material for such structures given its characteristic as a composite heterogeneous material of higher compressive strength, resistance to weathering, and good resistance to chemical attack; further, it is a generally low-maintenance material produced cheaply and with easily available raw materials [9,10,11,12,13]. However, it has a heterogeneous structure, so when exposed to high water pressure in sustained and/or repeated conditions, an understanding of its monotonic and cyclic responses is crucial.

Several research works have been devoted to investigating the influence of water pressure on concrete characteristics, such as compressive strength, peak strain, elastic modulus, and failure mode [23,27,28,29,30,31]. Most studies have been concerned with sensitivity to saturation conditions and the loading strain rate. Haynes et al. [18] conducted experiments on the effect of water pressure on concrete uniaxial static strength, wherein a reduction of 10% was observed in specimens subjected to 61 MPa of water pressure. Van der Wegen et al. [27] found an almost insignificant effect of high water pressure (10 MPa) on concrete strength. Clayton [30] reported reductions of 12% and 50% in compressive and flexural strength, respectively, of concrete after being subjected to 60 MPa of water pressure for six days. Hu et al. [31] experimentally examined the uniaxial compressive strength of concrete under both natural and saturated conditions, finding that greater strength is exhibited by concrete specimens in the water-saturated condition. Wang et al. [23] also concluded a nonlinear decrease in static compressive strength as the water pressure was increased up to 10 MPa.

Few studies have been conducted on the response of concrete to cyclic high water pressure, and the main concern has been on the mode of failure [23,26,32,33,34,35]. Chen et al. [34] carried out a first dynamic mechanical experiment with water pressure on concrete, wherein loading was applied when the water pressure was steady and the saturation condition of the specimen was not clarified. Poinard et al. [33] reported that the most severe cement matrix damage was observed between 60 and 150 MPa in a cyclic hydrostatic test, with linear elastic behavior observed for confining pressures less than 60 MPa. Under low confining pressure, failure was caused by crack propagation parallel to the primary load direction; however, cracks became less frequent and oriented in a sidewise direction under higher confinement [26,35]. Zhou et al. [32] concluded that the maximum reduction in static and dynamic compressive strength was 13.4% in testing under 4 MPa of water pressure. Given this limited understanding, further experiments on the effect of static and dynamic water pressure on concrete compressive strength are necessary that are not yet considered in the aforementioned literature, particularly considering deterioration related to the penetration of water into a specimen and migration within it.

The application of reinforced concrete for permanent and temporary deep ocean structures has recently become more prevalent, for strengthening and erection purposes. The static and dynamic high water pressure might significantly influence the bond strength between matrix and reinforcing bars. Although the authors have found no research into the effect of high water pressure on reinforcement bonding under static and cyclic conditions, they attributed that it influences the deterioration of the anchorage at the matrix/rebar interface. On the other hand, there have been a few studies on the fatigue response of reinforced concrete in the presence of water, which focused on the bond strength of deformed steel bars [36,37,38,39]. It was concluded that repeated reverse-cyclic loading led to pulverization of the concrete in the vicinity of the deformed steel bar, turning it into sludge and leading to pullout failure. Thus, it is clear that there is a need for experimental work on the effect of water pressure on reinforcement bond strength.

In this study, the aim is to comprehensively investigate the effect of sustained and cyclic high water pressure (60 MPa) on the mechanical properties of concrete, representing the applications of deep-sea structures while exposing it to various pressures during transportation and due to the tide effect. Concrete cylinders with and without an embedded steel bar were tested. Four series of specimens were tested having a uniaxial compressive strength of 58.1 MPa. In the first series, sustained high water pressure tests of 60 MPa were conducted on concrete cylinders without a steel bar for 7 and 60 days. In the other series, cyclic pressures at 60 MPa maximum water pressure were carried out on concrete cylinders with and without a steel bar for 10, 20, 30, 60, and 150 cycles. Regarding that, a high water pressure apparatus was designed with a limit capacity of 100 MPa. The residual compressive strength, bond strength, associated damage mechanism, degree of damage, and damage evolution were evaluated after exposure to high water pressure.

## 2. Experimental Program

The experimental program comprised four series of concrete cylinder specimens with and without a steel bar. These were tested under a sustained and repeated water pressure of 60 MPa. In the sections below, the material properties of the specimens, their number and geometry, the test apparatus, and the test setup are described.

### 2.1. Material Properties

The following materials were used for mixing the concrete: Ordinary Portland cement, fine aggregate, coarse aggregate, water, and superplasticizer. The water-to-cement ratio was designed to be 0.5, following the Japan Society of Civil Engineers (JSCE) standard [40] recommendation of less than 0.6 for watertight concrete. The composition of the concrete and its fresh properties are shown in Table 1. The ordinary Portland cement binding material had a specific gravity of 3.15 following the Japanese industrial standards (JIS). River sand of 2.65 specific gravity and 4.75 mm maximum aggregate size was used as fine aggregate. Crushed sandstone of 2.66 specific gravity and 13.0 mm maximum aggregate size was used as coarse aggregate. Furthermore, an air-entraining water-reducing agent (Master Pozzolith No. 70) was used as a superplasticizer to obtain an average slump value of 100 mm and an air content of 3.0% following the JSCE standard [41].

After casting, the specimens were kept in the molds and covered with plastic wrap for 24 h. They were then removed from the molds and cured by submerging in the water at a constant temperature of 22 ± 2 °C for 28 days using shallow containers to satisfy the full saturation condition. The concrete cylinder specimens measuring 100 mm in diameter and 200 mm in height had an average uniaxial compressive strength of 58.1 MPa after 28 days.

### 2.2. Test Apparatus (Water Pressure Device)

**Figure 1:**
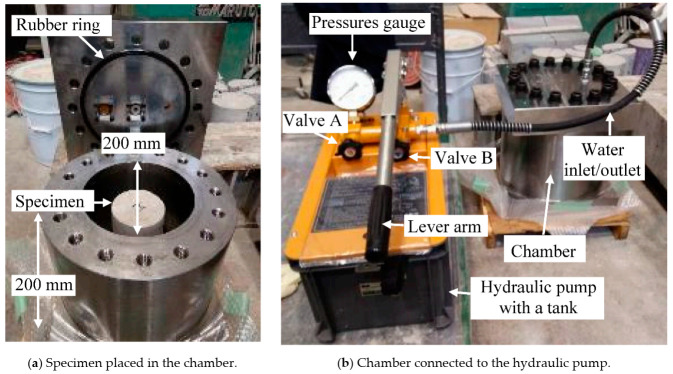
High-water-pressure apparatus.

A high water pressure apparatus was designed for the investigation of the effect of water pressure on the concrete cylinders, as shown in Figure 1. The maximum working water pressure was 60 MPa, simulating a 6000 m water depth at maximum for deep ocean RC stations in exploring the natural resources, and the apparatus has an ultimate capacity of 100 MPa water pressure. There are two main parts to the apparatus: A cylindrical pressure chamber with a diameter of 200 mm and a height of 200 mm, and a hydraulic pump. The latter consists of several components: A water-filled tank connected to the pressure chamber with a hose, a water inlet/outlet valve (valve A), a stop valve (valve B), a lever arm, and a pressure gauge.

To use the apparatus, a cylindrical concrete specimen is first placed in the center of the pressure chamber, and the chamber is then filled with water. The steel cover is placed on the chamber and tightened with bolts; a rubber sealing ring ensures pressure-tightness, as shown in Figure 1a. The chamber is pressurized by manually pumping water from the tank into the chamber using the lever arm, as shown in Figure 1b. The water pressure inside the chamber is controlled using the valves (A and B) and monitored with the pressure gauge. The water pressure can be quickly released using the valves (A and B), returning the pressurizing water to the tank through the hose.

### 2.3. Specimens and Testing Series

**Figure 2:**
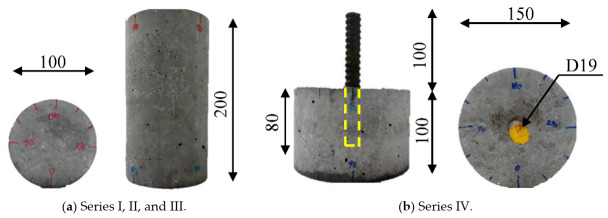
Specimen geometries (dimensions in mm).

Figure 2 shows the geometries of the concrete cylinders with and without an embedded steel bar that were chosen in the presented study for the desired four series. Figure 2a shows the standard cylinder geometry that was casted for series I, II, and III, having a diameter of 100 mm and a height of 200 mm, while the casted specimen for series IV was a cylinder with a diameter of 150 mm and a height of 100 mm, as shown in Figure 2b. A steel deformed bar with a nominal diameter of 19.1 mm was embedded inside the concrete cylinder, which is one of the most used reinforcements in the construction field, with an 80 mm embedded length (around four times the bar nominal diameter). The material properties of the embedded steel bar are shown in Table 2. The short embedment length of the steel bar was designed to avoid the yielding under pullout loading. The geometry of the tested specimen for series IV was designed to be fit inside the chamber of the testing apparatus.

Table 3 lists the specimens, giving the specimen layout, type of loading applied, history of the high-water-pressure test, and processing after testing for each series. Noting that, the permeability, porosity, and Scanning Electron Microscope SEM (manufacturer, city, country) analysis are not measured in the current study. In addition, the long-term resistance of concrete is not considered to be influenced by the hydration process, etc.; hence, this study focuses on only the mechanical properties. Two types of cylindrical specimens were tested. Those without an embedded steel bar are designated “PC” in Table 3 and Figure 2a. On the other hand, the concrete cylinders with an embedded steel bar are designated “RC”. Two testing conditions were investigated: Sustained water pressure of 60 MPa designated “D” and cyclic pressurization designated “R”. Regarding that, the tested specimens were classified and separated into four series, as listed in Table 3. Furthermore, control specimens of each type, labeled “PC” and “RC”, were subjected to a uniaxial compression test and a pullout test, respectively.

### 2.4. High-Water-Pressure Test Procedure

**Figure 3:**
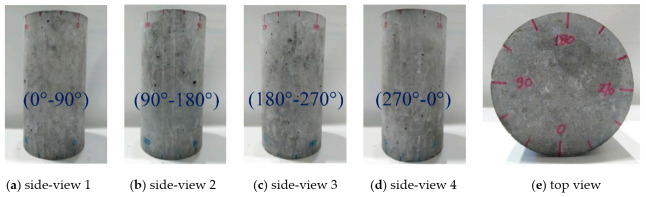
Marking of specimens.

All tested specimens were cured through submerging using shallow containers satisfying the full saturation condition for the desired period before testing. After curing, the surfaces of each specimen were dried to allow markings to be applied around the circumference, dividing the cylinder into four view locations. Figure 3 shows the marked top of a specimen and the four side views: 0–90°, 90–180°, 180–270°, and 270–0°. These markings allowed for the monitoring of the damage level and damage evolution in terms of crack location and width during and after testing. Each specimen (except PC and RC, which were not tested under high water pressure) was placed in the pressure chamber with the 0° marking near or parallel to the hose inlet.

The first series of specimens, namely I-PC-7D and I-PC-60D, were tested under sustained water pressure of 60 MPa for 7 and 60 days, respectively, before releasing the pressure. A repeated application of high water pressure was carried out for the second, third, and fourth series, with a water pressure gradually increased up to 60 MPa (at a rate of 0.05 MPa.s^−1^) and held for 10 min in each cycle, then immediately released to zero. This loading–unloading cycle follows the procedure proposed by Hori et al. [42], which aims to let the 60 MPa water pressure stabilize and penetrate the concrete pores. At the end of the water pressure test, each tested specimen was taken out and then kept at room temperature for 2 to 3 h until the surface dry condition was satisfied, with the aim of monitoring the surface cracks.

(1) $$Crack\;penetration\;depth\;\left(mm\right)=\frac{1}{2}of\;diameter\;of\;specimen-L_{c}$$

### 2.5. Processing after the High-Water-Pressure Test

After water pressure testing, specimens in the first and second series were subjected to a uniaxial compression test to measure the residual compressive strength of the concrete and assess their failure mode. The effect of sustained and cyclic applications of high water pressure was investigated by comparing the results with those for the control specimen (PC).

**Figure 4:**
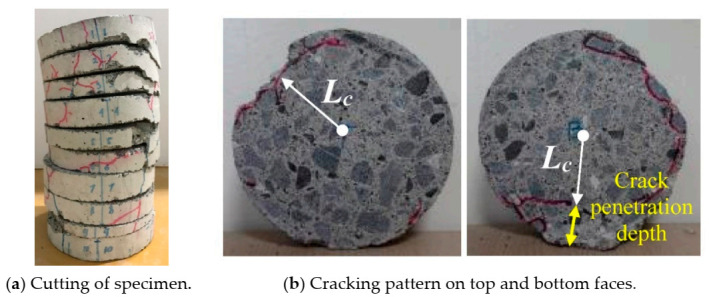
Measuring of crack penetration depth.

On the other hand, specimens in the third series were cut using a metal cutting saw. The cutting speed of the blade was less than 0.5 mm/s, slow enough to avoid the additional cracking. They were cut into ten slices by height after testing, in order to observe internal cracking for comparison with surface cracks, as shown in Figure 4a. Images of the internal cracking pattern for each slice on both the top and bottom faces were monitored, as shown in Figure 4b, with indications to illustrate the method of recording the maximum crack penetration depth. As can be seen, some chipping of the concrete occurred during the cutting process. Therefore, to obtain the crack penetration depth, the distance (*L_C_*) between the slice center and the point of deepest crack penetration was measured (see Figure 4b); then, the crack penetration depth was obtained using Equation (1). Finally, the surface and internal cracking conditions are compared for the two specimens (III-PC-60R and III-PC-150R) to gain an understanding of the fracture mechanism of concrete after repeated high water pressure.

**Figure 5:**
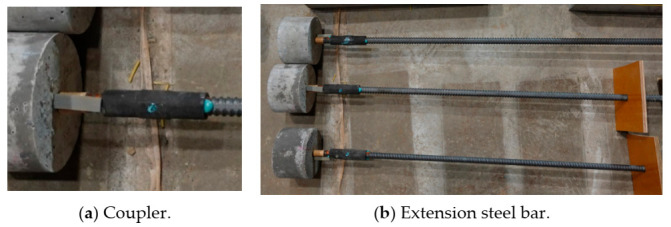
Pullout testing for series IV specimens.

**Figure 6:**
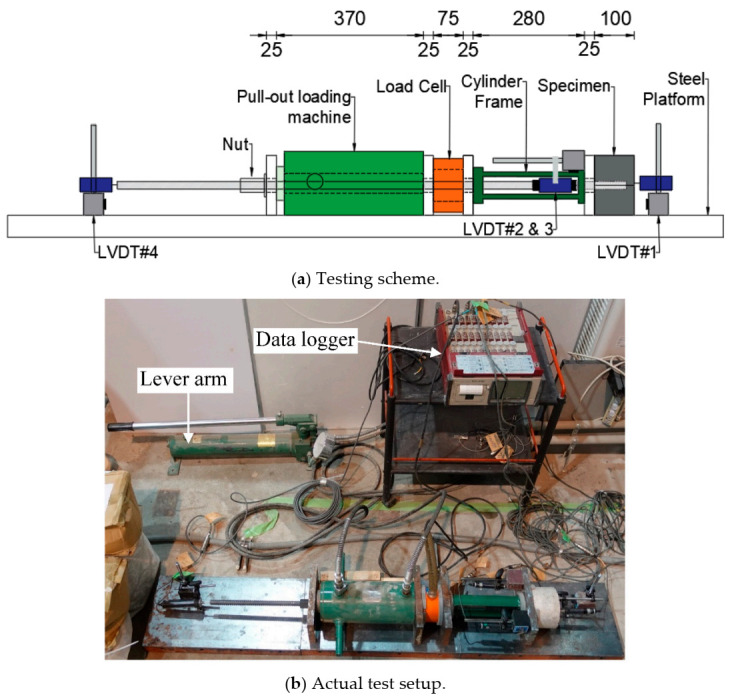
Pullout testing (dimensions in mm).

For the fourth series of specimens, which were the concrete cylinders with an embedded steel bar, pullout tests were implemented to estimate the residual pullout capacity and evaluate the degree of bond damage at the matrix/steel bar interface. To carry out the pullout test, a long steel extension bar with a nominal diameter of 19.1 mm and a length of 110 cm was securely connected to the embedded bar using a coupler, as shown in Figure 5. This gave the loading jack sufficient purchase on the steel bar to apply tensile force for the pullout, as could be seen in Figure 6.

Figure 6 shows the pullout test setup, which follows the same procedure as used in Punyawut et al. [43], wherein the applied tensile load and the associated displacement are measured using a load cell and linear variable differential transformers (LVDTs) (Tokyo Measuring Instruments Lab., Tokyo, Japan), respectively. The relative displacement of the steel bar from the concrete is calculated from the average displacement obtained from two LVDTs, LVDT#2 and LVDT#3. Besides, any movement of the concrete specimen and the extension steel bar was monitored by fitting them with LVDT#1 and LVDT#4. Pullout capacity was measured using Equation (2).

(2) $$Pullout\;capacity\;\left(MPa\right)=\frac{P_{max}}{A}=\frac{P_{max}}{\pi \cdot D\cdot L_{er}}$$

where *P_max_* is the maximum applied tensile load (*N*), *D* is the steel bar nominal diameter (mm), and *L_er_* is the length of the steel bar embedded in concrete, which is around 80 mm.

## 3. Results and Discussions

### 3.1. Series I: Sustained High-Water-Pressure Test

**Figure 7:**
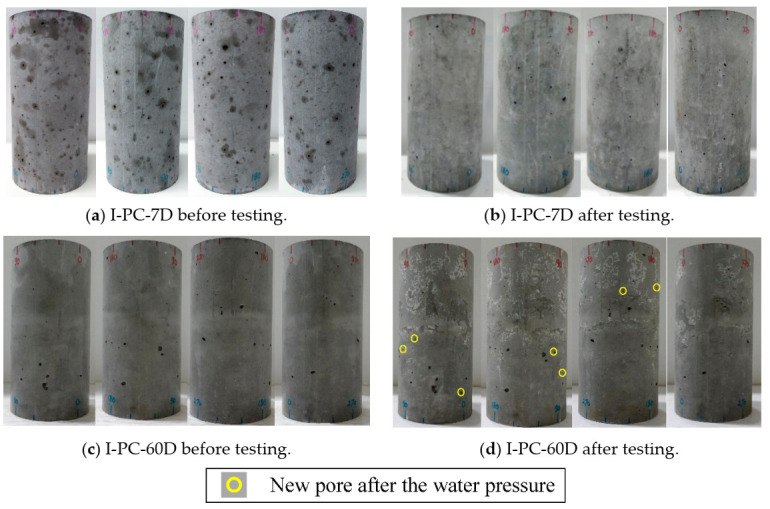
Specimen surface condition for series I under sustained high water pressure.

Figure 7 shows the surface cracking for the four side views of specimens I-PC-7D (7 days under pressure) and I-PC-60D (60 days under pressure) obtained before and after sustained high-water-pressure testing at 60 MPa. In the case of I-PC-7D, there is no change in pores, cracks, or damage after 7 days of high water pressure as compared with that before testing, as shown in Figure 7a,b. On the other hand, specimen I-PC-60D after 60 days of high water pressure has small pores on the surface but no cracking. These pores are marked using yellow rings in Figure 7c,d. These seven marked pores may have occurred because of the washing out at weaker points of the concrete surface because the specimen was maintained in a fully saturated condition.

**Figure 8:**
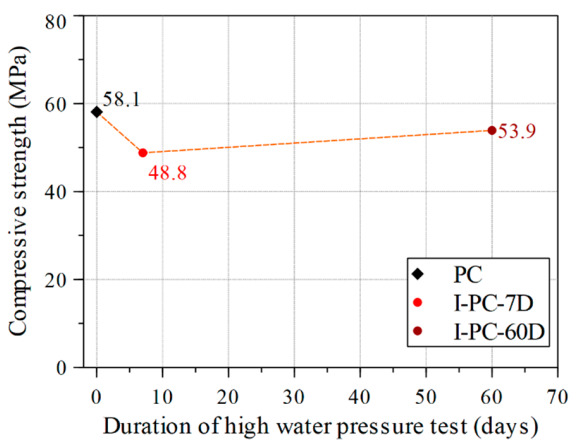
Compressive strength versus duration under sustained high water pressure.

After these concrete cylinders had been tested under sustained high water pressure of 60 MPa (which is about the same as their initial uniaxial compressive strength), a uniaxial compression test was conducted to estimate the residual compressive strength. Figure 8 shows the results for the two specimens as compared with the control specimen (PC). An instant reduction in compressive strength by around 16% occurred after 7 days under pressure. However, after 60 days of sustained high water pressure, the compressive strength had stabilized and recovered slightly compared with the value at 7 days. The marked reduction in strength early in the application of sustained high water pressure highlights the role of water flow driven by the differential between internal and external water pressure. The differential leads to active changes in pore water pressure that cause local stresses, and these eventually result in the loss of concrete strength, as previously reported by Clayton [30].

### 3.2. Series II: Repeated High-Water-Pressure Test

**Figure 9:**
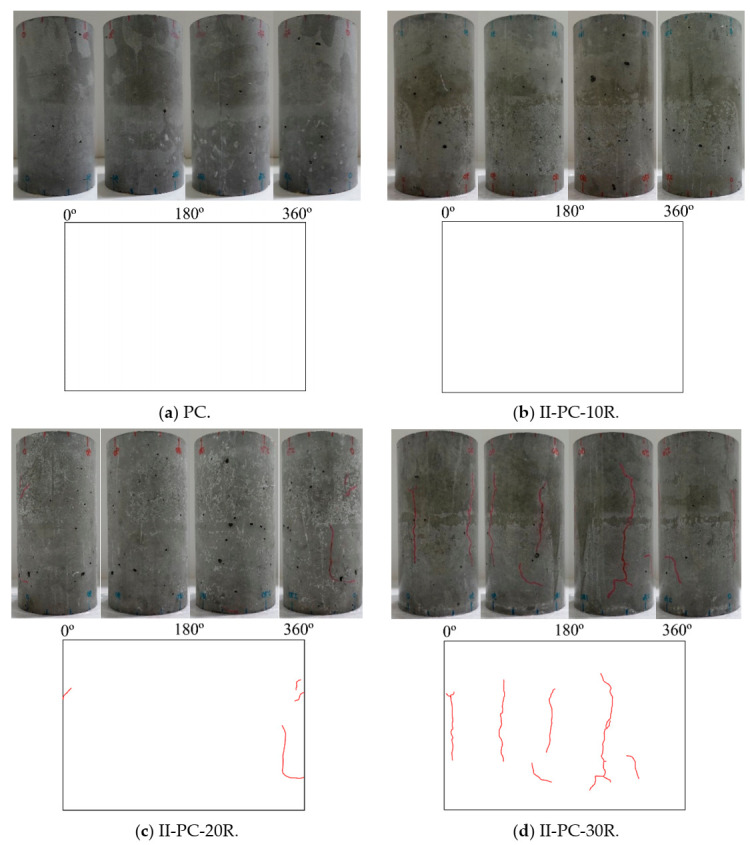

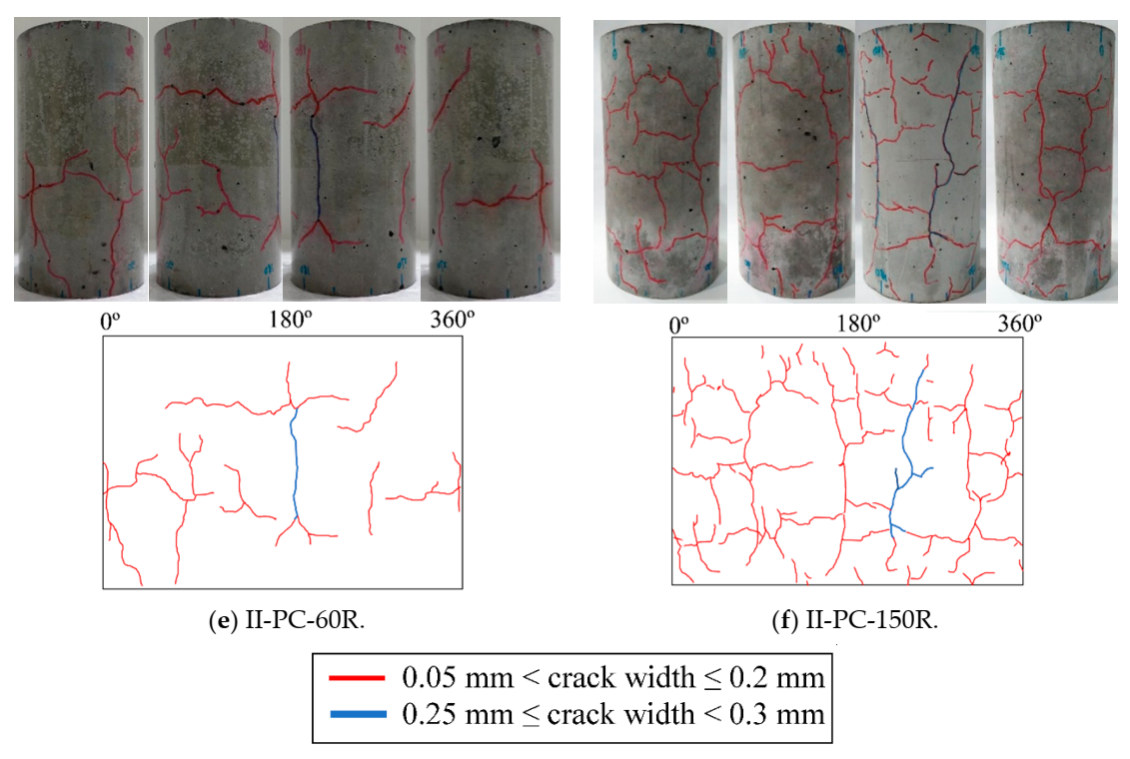
Specimen surfaces of series II specimens subjected to repeated high water pressure.

Figure 9 shows the surface cracking of tested specimens after the repeated application of high water pressure for 10, 20, 30, 60, and 150 cycles. Cracks ranging in width between 0.05 and 0.20 mm are colored red, with blue used for crack widths ranging between 0.25 and 0.30 mm. It can be seen in this figure that, for specimen II-PC-150R, cycles of loading and unloading resulted in gradually propagating cracks on the surface up to 150 cycles. After the initiation of the cracks, they gradually joined together in the lateral and longitudinal direction, resulting in more severe damage as the number of cycles increased.

**Figure 10:**
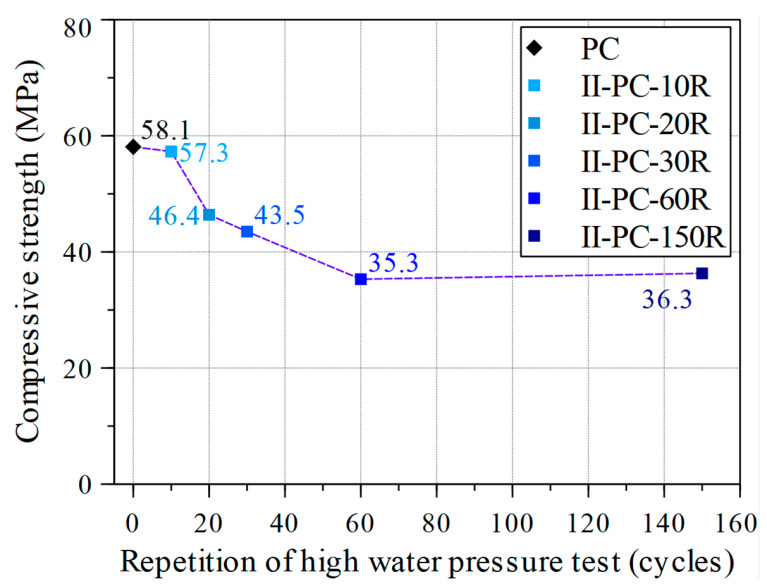
Compressive strength versus the number of repeated cycles of high water pressure.

After testing these concrete cylinders under cyclic high water pressure at the maximum load of 60 MPa, a uniaxial compression test was conducted to investigate residual uniaxial compressive strength. Figure 10 shows the results of this residual uniaxial compressive strength test for specimens II-PC-10R, II-PC-20R, II-PC-30R, II-PC-60R, and II-PC-150R as compared with the control specimen (PC). With no observed surface cracks on specimen II-PC-10R in Figure 9b, its residual compressive strength was almost no different from the 57.3 MPa of the control specimen. After that, with the increase in the number of cycles, the residual compressive strength gradually decreased up to 40% (35.3 MPa) in the case of the 60-cycles specimen, accompanied with a simultaneous propagation of surface cracks, as shown in Figure 9c–e. Although cracking continued to evolve between specimens II-PC-60R and II-PC-150R, as seen in Figure 9e,f, the residual compressive strength did not change between 60 and 150 cycles, remaining at almost 36.3 MPa. This phenomenon is observed in more detail in the third series, where the aim was to visually observe and understand the internal cracking state of specimens subjected to 60 and 150 cycles of repeated high water pressure by cutting the specimen into ten slices.

**Figure 11:**
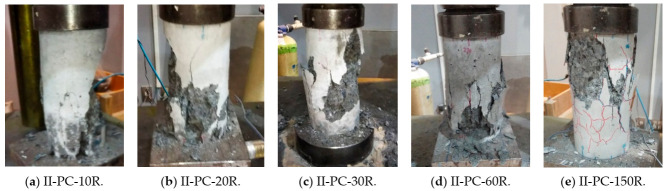
Failure modes of series II specimens under uniaxial compression.

Finally, Figure 11 shows the failure modes in the uniaxial compression loading tests, where all specimens exhibited a shear mode of failure. Even though specimens II-PC-60R and II-PC-150R had severe surface cracks, their failure mode was shear, indicating that a strong core remained regardless of the surface cracks.

### 3.3. Series III: The Internal Crack Condition of the Tested Specimen

**Figure 12:**
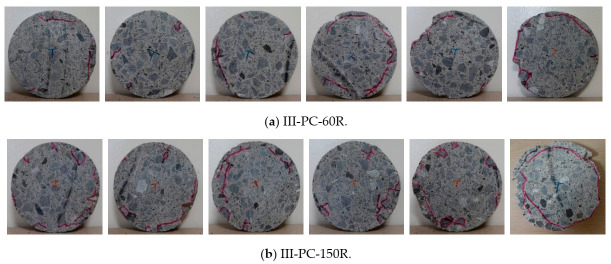
Internal cracking state for typical slices ranging from No. 4 to No. 9 of series III after repeated high-water-pressure cycles.

Aiming at checking the internal cracking state, concrete cylinder specimens that had been subjected to cyclic high water pressure for 60 cycles (III-PC-60R) and 150 cycles (III-PC-150R) were cut into 20 mm slices in the height direction, as shown in Figure 4a. Figure 12 shows the internal cracking state on the top and bottom faces of some of these concrete slices. Table 4 lists the maximum crack penetration depth in each slice for both tested specimens as calculated using Equation (1). It is clear that even though the maximum penetrating crack is located in different slices of the two specimens, the maximum crack depth in both cases is 25 mm. That is, the further penetration of cracks halted at a certain depth (25 mm) between 60 and 150 cycles. Crack propagation then proceeded parallel to the cylinder surface to form a circular crack, as noted in Figure 12.

**Figure 13:**
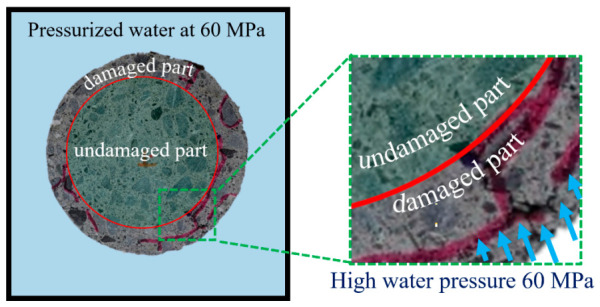
Penetration of the pressurized water inside the concrete specimen depth.

Figure 13 illustrates the penetration of pressurized water at 60 MPa into the concrete cylinder after a certain number of loading–unloading cycles between 60 and 150 cycles. An undamaged core of concrete remains without any cracks, while cracks propagate around the damaged zone as cracks within this weaker zone join together. There is a clear tendency for cracks to form and develop within the already damaged outer region than to penetrate deeper inside the undamaged zone.

This might explain the similar residual compressive strength and shear failure mode of specimens II-PC-60R and II-PC-150R in Figure 10 and Figure 11. Although II-PC-150R exhibits more severe surface cracking than II-PC-60R, the maximum crack penetration depth of both is similar, so they have the same undamaged concrete core resulting in a stable residual strength. It could be concluded that the residual compressive strength and mode of compression failure are strongly related to the crack penetration process and the associated core of undamaged concrete, while the observed surface cracking is of less importance.

### 3.4. Series IV: Pullout Strength of Tested Specimens

**Figure 14:**
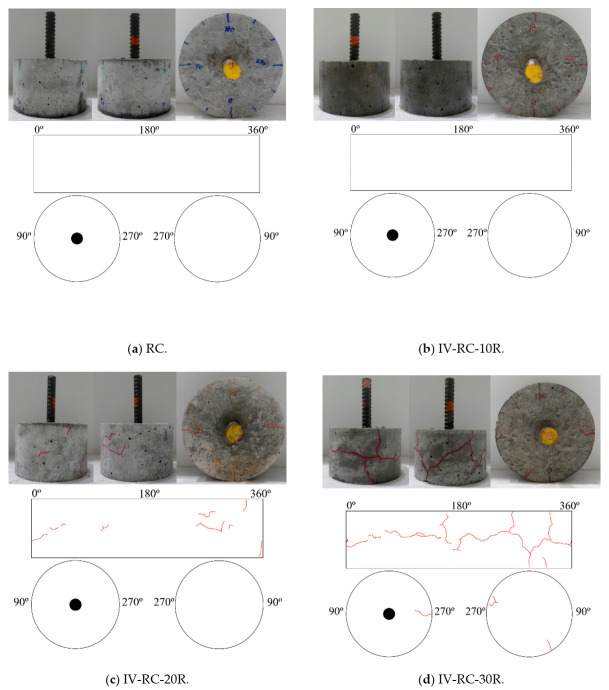

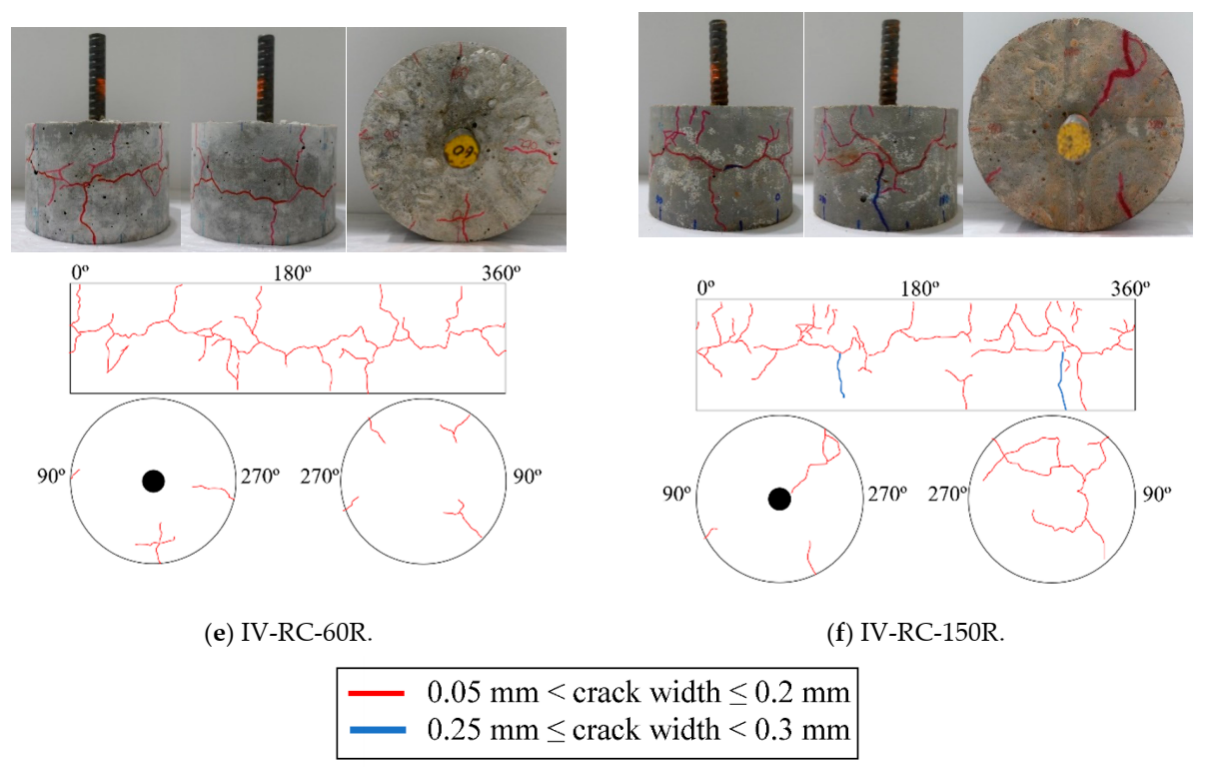
Specimen surface cracking for series IV after repeated cycles of high water pressure.

Figure 14 shows the surface cracking of cylinder specimens with an embedded steel bar after the application of repeated cycles of high water pressure at 60 MPa maximum pressure. After 10, 20, 30, 60, and 150 cycles, the surface cracking is markedly similar to that in the second series. In this figure, the cracks are similarly colored red for widths between 0.05 and 0.20 mm, and blue for widths between 0.25 and 0.30 mm. Surface cracking becomes visible after 20 loading–unloading cycles (specimen IV-RC-20R) and cracks gradually become larger and longer in both radial and longitudinal directions up to 150 cycles. Seen from the top face of the specimen (where the steel bar protrudes), the propagation of cracks in the depth direction advances until they almost reach the embedded steel bar in specimen IV-RC-150R, as shown in Figure 14f.

**Figure 15:**
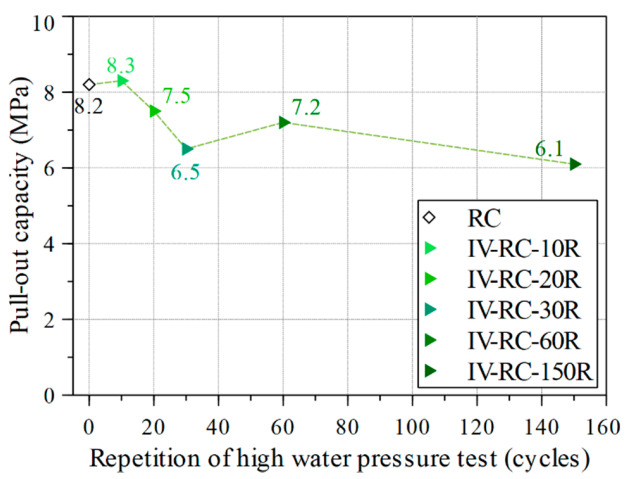
Pullout capacity versus the number of cycles of high water pressure.

After subjecting these concrete cylinder specimens to cyclic high water pressure, pullout tests were conducted to evaluate the residual pullout capacity, as explained in Section 2.5. Figure 15 shows the results for specimens IV-RC-10R, IV-RC-20R, IV-RC-30R, IV-RC-60R, and IV-RC-150R as compared with the control specimen (RC). After 10 loading–unloading cycles (specimen IV-RC-10R) and with no surface cracks observed (see Figure 14b), the residual pullout capacity was unchanged. Thereafter, residual pullout capacity gradually fell by 26% to 6.5 MPa from 10 to 30 cycles, as shown in Figure 14c,d. This corresponds with the propagation of surface cracks on the specimens. In specimens subjected to 60 and 150 cycles (IV-RC-60R and IV-RC-150R), the residual pullout capacity did not fall further but stabilized somewhat (see Figure 15), corresponding to the stabilization of compressive strength in the second series.

**Figure 16:**
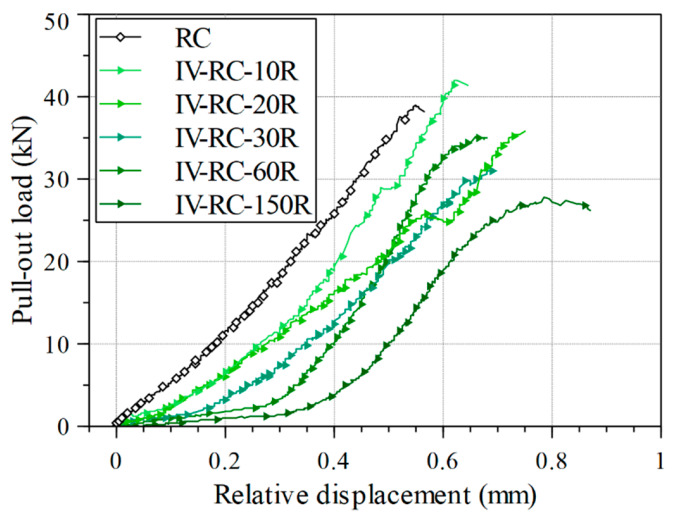
Pullout load versus steel bar relative displacement during pullout test.

Figure 16 shows the relationship between pullout load and relative displacement for the fourth series during the pullout tests. The results showed a degradation process in the stiffness, defined as the initial elastic slope of the load–displacement relationship in Figure 16, between 10 to 150 loading–unloading cycles of high water pressure. Further, there is an initial increase in relative displacement as loading starts, and the displacement increment is greater with more cycles. This might indicate slippage of the steel bar reflecting the loss of bond strength at the matrix/steel bar interface after repeated exposure to high water pressure.

**Figure 17:**
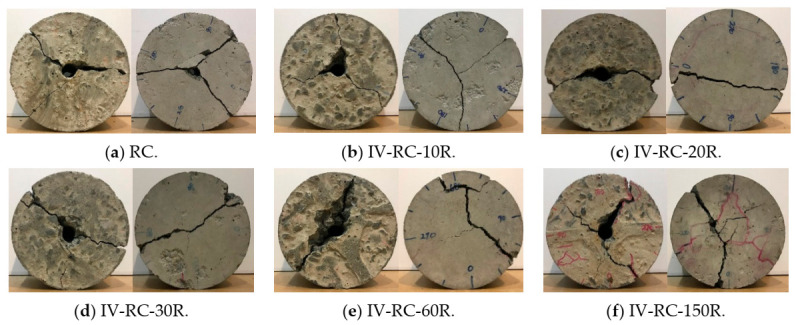
Failure state on top and bottom faces of series IV specimens after pullout test.

Figure 17 shows the condition of all specimens in the fourth series at the end of the pullout test. In each case, a splitting mode of failure occurs at the peak pullout load, with the specimen splitting into two or three parts. At this point, the specimens were opened up for the examination of the state of the interface between the steel bar and concrete.

**Figure 18:**
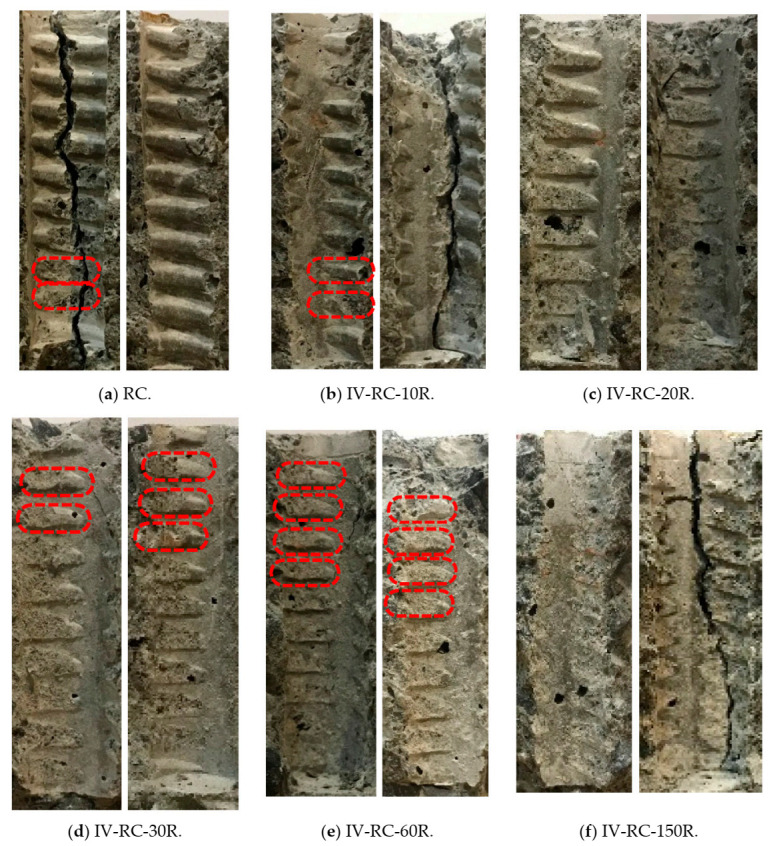
Matrix/steel bar interface for series IV specimens after pullout test.

Figure 18 shows the interface for each of these specimens, showing damage to the concrete ribs (an indicator of bond strength). Damage accrued gradually from 10 to 150 loading–unloading cycles, with the concrete finally becoming smooth and fully crushed in specimen IV-RC-150R after 150 cycles, as shown in Figure 18f. The repeated application of the high water pressure would have resulted in significant pore water pressure stresses, and these were particularly severe toward the top of the specimen near the steel bar. The result was incremental damage to the concrete ribs as the number of cycles increased. However, concrete being heterogeneous, the internal damage at the interface is not equally distributed; the red markings in Figure 18 indicate some ribs that are partially crushed but with some stronger parts intact. That could be the cause of the little increase in the residual pullout capacity for specimens IV-RC-10R and IV-RC-60R, as shown in Figure 15.

## 4. Conclusions

In this investigation of the effect of sustained and repeated application of high water pressure (60 MPa) to concrete cylinders with and without an embedded steel bar, the main degradation parameters are found to be fracture behavior, residual compressive strength, failure mode, and residual bond strength. The following conclusions have been drawn from the experimental work.

- The residual uniaxial compressive strength of concrete decreased immediately by 16% after 7 days of sustained high water pressure (60 MPa), but no surface cracks were observed. However, no further significant reduction in residual compressive strength was observed, as the period of sustained water pressure was extended to 60 days, although the formation of some surface pores was noted.
- The repeated application of high water pressure for 10 to 150 cycles caused inevitable damage to specimens as the propagation of surface cracks advanced. A significant reduction in residual uniaxial compressive strength reaching 40% was observed. Although all specimens exhibited a shear mode of failure in uniaxial compression tests, there was no further reduction in residual compressive strength after 60 loading–unloading cycles.
- The internal cracking state in specimens subjected to 60 and 150 cycles of high water pressure permitted a correlation with residual strength and surface cracking. The internal cracks were found to propagate within a certain damage zone as cracks within this weaker zone gradually joined as more cycles of loading were applied, leaving the core part of the cylindrical specimens in an uncracked state.
- Repeated cycles of high water pressure caused severe damage to concrete specimens with an embedded steel bar, as surface cracks propagated toward the steel bar and ultimately caused the loss of bond strength at the matrix/steel bar interface. This bond damage eventually led to a reduction of up to 36% in residual pullout capacity. In pullout tests, all specimens exhibited a splitting mode of failure.
- Bond damage in the reinforced specimens was examined by observing the condition of the concrete ribs at the interface between the matrix and steel bar after the pullout. Damage to the ribs intensified as the number of cycles increased. The result was a decrease in pullout stiffness and an increase in slippage of the steel bar from an early stage of loading.
- The detailed investigations of material properties such as permeability, porosity, SEM investigation, and long-term hydration effect on the concrete regarding the effect of repeated high water pressure are not considered in the current study. This information would be beneficial to reveal the deterioration mechanism in the future.

## Figures and Tables

**Table 1:** Mix proportions and fresh properties of concrete.

Water (kg/m^3^)	Cement (kg/m^3^)	Sand (kg/m^3^)	Gravel (kg/m^3^)	SP (kg/m^3^)	Slump (mm)	Air Content (%)
185	370	832	1021	0.2	100	3.0

**Table 2:** Material properties of steel bar.

Type	Diameter (mm)	Area (mm^2^)	Weight(kg/m)	Yield Strength (MPa)	Modulus of Elasticity (GPa)
D19	19.1	286.5	2.25	420	190

**Table 3:** Specifications of specimens for the high-water-pressure test.

Series No.	Specimen ID	No. of Specimens	Specimen Layout	High-Water-Pressure Test		Post-Test Processing
				Testing Condition	Testing History	
-	PC	3	Concrete cylinder	Control		Uni-axial compression test
I	I-PC-7D	1		Sustained	7 days	
	I-PC-60D	1			60 days	
II	II-PC-10R	1		Repeated	10 cycles	
	II-PC-20R	1			20 cycles	
	II-PC-30R	1			30 cycles	
	II-PC-60R	1			60 cycles	
	II-PC-150R	1			150 cycles	
III	III-PC-60R	1			60 cycles	Cutting into ten slices in height
	III-PC-150R	1			150 cycles	
-	RC	1	Concrete cylinder with an embedded steel bar	Control		Pullout test
IV	IV-RC-10R	1		Repeated	10 cycles	
	IV-RC-20R	1			20 cycles	
	IV-RC-30R	1			30 cycles	
	IV-RC-60R	1			60 cycles	
	IV-RC-150R	1			150 cycles	

**Table 4:** Crack penetration depth measurements.

Slice Number	III-PC-60R		III-PC-150R	
	Top	Bottom	Top	Bottom
No.1	-	8	-	6
No.2	8	10	15	13
No.3	13	11	13	13
No.4	13	13	16	16
No.5	12	9	21	21
No.6	15	15	18	25
No.7	19	19	20	16
No.8	21	25	15	15
No.9	15	14	17	17
No.10	8	-	14	-

## Data Availability

The authors declare that all data and methods used in the research are presented in sufficient detail in this paper so that other researchers can replicate the work including all raw data.
